# Photo-Electrochemical Treatment of Reactive Dyes in Wastewater and Reuse of the Effluent: Method Optimization

**DOI:** 10.3390/ma7117349

**Published:** 2014-11-14

**Authors:** Mireia Sala, Víctor López-Grimau, Carmen Gutiérrez-Bouzán

**Affiliations:** Institut d’Investigació Tèxtil i Cooperació Industrial de Terrassa (INTEXTER), Universitat Politècnica de Catalunya-BarcelonaTech (UPC), C/Colom 15, 08222 Terrassa, Spain; E-Mails: mireia@fitex.es (M.S.); victor.lopez-grimau@upc.es (V.L.-G.)

**Keywords:** electrochemical, UV irradiation, indirect-oxidation, effluents reuse, reactive dyes, mono-functional, bi-functional, tri-functional

## Abstract

In this work, the efficiency of a photo-electrochemical method to remove color in textile dyeing effluents is discussed. The decolorization of a synthetic effluent containing a bi-functional reactive dye was carried out by applying an electrochemical treatment at different intensities (2 A, 5 A and 10 A), followed by ultraviolet irradiation. The combination of both treatments was optimized. The final percentage of effluent decolorization, the reduction of halogenated organic volatile compound and the total organic carbon removal were the determinant factors in the selection of the best treatment conditions. The optimized method was applied to the treatment of nine simulated dyeing effluents prepared with different reactive dyes in order to compare the behavior of mono, bi, and tri-reactive dyes. Finally, the nine treated effluents were reused in new dyeing processes and the color differences (DE_CMC (2:1)_) with respect to a reference were evaluated. The influence of the effluent organic matter removal on the color differences was also studied. The reuse of the treated effluents provides satisfactory dyeing results, and an important reduction in water consumption and salt discharge is achieved.

## 1. Introduction

The textile industry produces large volumes of wastewater in its dyeing and finishing processes. These effluents have as common characteristic their high coloration. This work is focused on reactive dyes, mainly used in the cotton industry. They were selected because of their high consumption, which implies an environmental and aesthetic problem caused both in dyeing and soaping effluents. The conventional biological plant treatments are not effective in color removal because reactive dyes have aromatic rings in their large molecules that provide them chemical stability and resistance to the microbiological attack [1]. Although the effluent color regulations are very variable depending on the country, the biological treatments are insufficient to remove color and to accomplish with current regulations. Consequently, the application of tertiary treatments is required.

Several methods are used for the removal of organic dyes from wastewaters. The current physic-chemical methods, based on the separation between dye and effluents, produce a residue which requires an additional treatment to be destroyed. In addition, absorbent materials (such as active carbon, silica gel or alumina) require regeneration after several treatments [2]. The use of low cost adsorbents and natural adsorbents obtained from agricultural waste can avoid this expensive regeneration process [3,4,5]. The filtration and membranes methods need thorough chemical cleaning treatments to achieve a good performance [6,7]. Chemical oxidation methods are rather expensive and involve some operational difficulties [8,9]. Enzymatic decomposition requires further investigation in order to know which enzymatic process takes place [10]. Moreover, temperature and pressure have to be controlled to avoid enzyme denaturalization. For these reasons, the electrochemical methods are nowadays the subject of a wide range of investigations at laboratory and pilot plant scale. The advantage of the electrochemical techniques is that the electron is a clean reagent. They also have good versatility and high energy efficiency. They are safe and easy for automation, because it is possible to operate at smooth conditions [11]. On the other hand, one of the main disadvantages of electrochemical treatments is their high energy cost which can be reduced by indirect electrochemical treatments in the presence of chloride.

As some industrial wastewaters contain large amounts of chloride, the indirect electrochemical oxidation method with active chlorine is the most suitable technique to treat this kind of effluent. The addition of chemical products in the electrolysis is not required because chloride is used as an electrolyte. In contrast, the combination of electrochemistry and chloride can produce haloforms such as chloroform, although it is not an inconvenience if the treated water is degraded lately in a biological plant to accomplish its mineralization. In fact, it has been verified that the concentration of haloforms is very low and they do not show any toxic effect on the plant microorganisms [12]. Moreover, the combination of UV exposure with the electrochemical treatment (photo-electrochemical methods) achieves the decomposition of the small amount of haloforms generated during the electrochemical treatment [13].

The electrochemistry method using chlorine as indirect oxidant has been noted to be effective in several kind of dyes, such as azo dyes [14], acid dyes [15] or disperse dyes [16] and combined with photo-electrochemistry has also provided good results for phtalocyanine dyes degradation [17], but in this case, the metal ions liberated (*i.e.*, copper) have to be removed.

The indirect electro-oxidation occurs when strong oxidants are generated *in situ* during the electrolysis and react with the organic pollutants such as dyestuffs, resulting in their total or partial degradation.

In the electro-oxidation with active chlorine [18,19] (which is a major oxidizing agent), the free chlorine gaseous and/or the generated chlorine-oxygen species such as hypochlorous acid (HClO) or hypochlorite ions (ClO^−^) depending on the pH, oxidize the organic matter present in the effluents, according to the reactions (1)–(3):

2Cl^−^ → Cl_2(aq)_ + 2e^−^ (1)

Cl_2(aq)_ + H_2_O → ClO^−^ + Cl^−^ + H^+^ (2)

Dye + ClO^−^ → intermediate compounds → CO_2_ + H_2_O + Cl^−^ (3)

Moreover, the current policies concerning water and energy consumption are conducive to recycling and reuse treatments. In this sense, recent studies demonstrate the possibility of reusing these discolored effluents for new dyeing processes. The reuse of 70% of discolored dyebaths, after electrochemical treatment assisted by UV irradiation, provides in most of cases, low color differences (DE_CMC (2:1)_ ≤ 1, which is the limit for acceptance in the textile industry) with respect to the original dyeing with decalcified tap water.

Most electrochemical decolorization studies are focused on reactive dyes. They represent about 20%–30% of the total market [20], because of their washing fastness and brilliant color. Their structure contains one or several *reactive groups* (which react with the fiber) and a *chromophore group* (which gives the color). The most used chromophore group is the “azo” (R–N=N–R’), followed by the anthraquinone group [21]. Azo dyes constitute more than half of worldwide production [22], approximately 65% [23,24,25].

In the case of triazine reactive dyes, there is a dyeing reaction with the fiber that occurs by a nucleophilic displacement of the substituent atom from the reactive group of the dye (*i.e.*, chlorine) to the hydroxyl group from the cellulose in alkaline medium, according to the reaction (4) [26].

dye-X + HO-cellulose → dye-O-cellulose + HX(4)

Simultaneously, there is a competitive reaction between dye and water which produces dye hydrolysis (reaction (5)). The hydrolyzed dyes cannot react with the fiber, and this is the major cause of the effluent color.

dye-X + H_2_O → dye–OH + HX (5)

Initially, reactive dyes contained only one functional group in their molecule. However, since the 1970s there has been an increase of the number of functional groups. Thus, reactive dyes can be classified as mono, bi, or tri-functional depending on the number of groups able to react with the fiber. The dye exhaustion is related to this factor. The exhaustion value for mono, bi, tri-reactive dyes goes from 60% to 90%. As it is expected, the bi- and tri-reactive dyes react faster with the fiber and consequently, they achieve higher dyeing exhaustion (which implies less dyes hydrolysis).

This work was structured in two parts. In the first one, the study was carried out with the bi-functional dye Procion Navy HEXL (referred to as PN): different intensities were applied to a synthetic dyeing effluent, defining their kinetic rate order. Then the optimization of the photo-electrochemical method was carried out with the appropriate intensity.

The second part of this paper is based on the application of the optimized photo-electrochemical method to eight additional reactive dyes (mono-, bi- and tri-functional), with the aim to obtain their kinetic rates. Subsequently, the reuse of the treated effluents was studied. In all cases, 70% of the dyeing wastewater was treated and reused, as 30% remains on the fabric. Finally, the influence of the total organic matter content on the dyeing obtained with the treated effluent was studied.

Consequently, the main goal of this study is to optimize the photo-electrochemical method for the treatment of dyeing effluents and their reuse in a new dyeing process, with the aim to reduce the consumption of water and the discharge of salts.

## 2. Results and Discussion

### 2.1. Electrochemical Treatment: Effect of the Intensity

**Figure 1:**
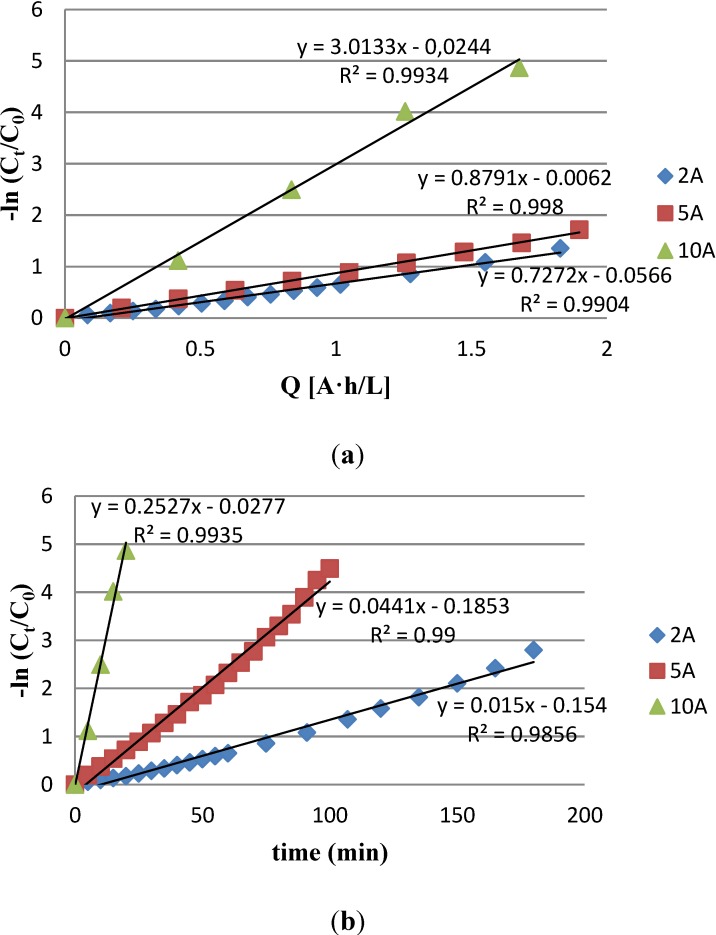

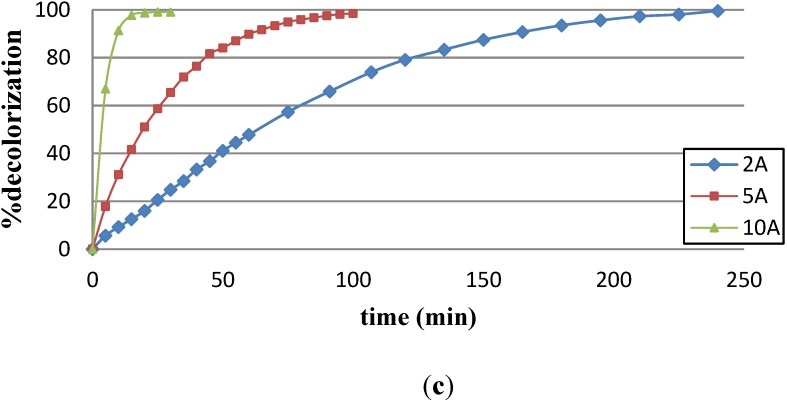
Procion Navy HEXL (PN) Decolorization with electrochemical treatments at 2 A, 5 A and 10 A. (**a**) PN kinetic rate in time; (**b**) PN kinetic rate in applied charge; (**c**) Evolution of PN decolorization.

The electrochemical kinetic rate degradation of PN dye was studied at three intensities (2 A, 5 A and 10 A). Results are plotted in Figure 1.

The representation of −ln(C_t_/C_0_) *vs.* time (Figure 1a) showed that in all the studied cases, the dye degradation follows a first-order reaction kinetics (see Section 3.5.2, Equations (7) and (8)). As expected, the treatment time required to obtain a specific degradation was shorter when the intensity applied was higher. In order to know the efficiency of the treatments, −ln(C_t_/C_0_) *vs.* the specific charge applied (A·h/L), which is a normalized parameter, were plotted in the Figure 1b. The results showed that the slopes at 2 A and 5 A were of the same order (0.73 and 0.88 respectively). In this case, the time required to achieve a fixed decolorization had an inverse linear relationship with the intensity (at constant volume). On the other hand, when the intensity is increased to 10 A, the slope becomes much higher. The time necessary to obtain the fixed decolorization is much lower than expected. Probably this higher current value promotes the generation of additional oxidant species produced from chloride oxidation (such as Cl_2_, OCl^−^ and chlorine radicals) or produced from water oxidation, such hydroxyl radical (•OH), atomic oxygen (•O), hydrogen peroxide or ozone [27].

Decolorization values of 99% can be reached with all the studied intensities (Figure 1c). The treatments at 2 A and 5 A requires a similar power consumption (29.70 W·h and 28 W·h respectively), whereas at 10 A, the consumption is reduced to 15.17 W·h to achieve the same decolorization rate. The power consumption was calculated from the corresponding voltage value for each intensity value (5.4 V, 5.2 V and 9.1 V at 2 A, 5 A and 10 A respectively). The obtained power values were multiplied by the time of treatment in hours.

Consequently, it can be stated that the decolorization treatment performed at 10 A is more efficient according to the kinetic rate, the specific charge applied and power consumption results. Therefore, the subsequent studies were carried out at this intensity.

### 2.2. Optimization of the Photo-Electrochemical Treatment for PN at 10 A

When an electrochemical treatment is applied to a solution which contains chloride and organic matter, small amounts of halogenated volatile compounds can be generated, mainly haloforms. In order to solve this problem, UV radiation is applied. Several studies were carried out by combining the electrochemical treatment with UV radiation. Table 1 shows the results corresponding to the final decolorization achieved and the decolorization kinetic rate, for electrochemical treatment (trial A) and the different combination of electrochemical and UV treatment studied on the PN effluent (trials B–E).

The results shown in Table 1 indicate that the electrochemical treatment was the faster technique to achieve 99% of decolorization because its kinetic rate was higher. When UV radiation was applied simultaneously with the electrochemical treatment (UVEC), 99% decolorization was also achieved but the kinetic rate decreased due to the UV light degradation of the indirect oxidant compounds, generated during the electrolysis (HClO/ClO^−^). On the other hand, it can be appreciated that the UV radiation after the electrochemical treatment enhances the dyes decolorization.

Taking into account the local regulations on discharge limits, the full decolorization is generally not required. In fact, it is commonly established that no color must be appreciated when the effluent is diluted 1/10–1/30 (this value change depending on the Country and even of the different regions). Then, the more appropriate selection is an initial electrochemical treatment followed by the UV irradiation.

Among the combinations of these techniques, 5 min of electrochemical treatment provided decolorization yields around 57%. If the treatment was continued with the combined treatment UVEC, 99% of decolorization would also be achieved, whereas 66% is obtained with the UV radiation alone. For that reason, the authors decided to follow up the electrochemical treatment during 10 min (80% of decolorization) with further UV radiation to achieve 86% decolorization.

**Table 1:** Characterization of the electrochemical and photo-electrochemical treatments in kinetic rate, total organic carbon (TOC) removal and decolorization, applied to PN at 10 A.

Trial	Treatments	Decolorization (%)		Kinetic rate (min^−1^)		*R*^2^	
		Treat 1	Treat 1 + 2	K_1_	K_2_	K_1_	K_2_
A	EC	99	-	0.2729	-	0.99	-
B	UVEC	99	-	0.1332	-	0.99	-
C	(1) 5 min EC	55	-	0.2715	-	0.99	-
	(2) UVEC		99		0.1598		0.99
D	(1) 5min EC	59	-	0.2738	-	0.99	-
	(2) UV		66		0.0014		0.97
E	(1) 10 min EC	80	-	0.2722	-	0.99	-
	(2) UV		86		0.0037		0.98

Where: EC = electrochemical treatment. UVEC = electrochemical treatment with simultaneous UV radiation. EC + UV = electrochemical treatment with posterior UV radiation. K_1_: kinetic rate corresponding to treatment 1. K_2_: kinetic rate corresponding to treatment 2.

In this section, the kinetic rates expressed in charge or in time units will have the same evolution because all the experiments were run at the same intensity (10 A). Consequently, they were only reported in time units (min^−1^).

**Figure 2:**
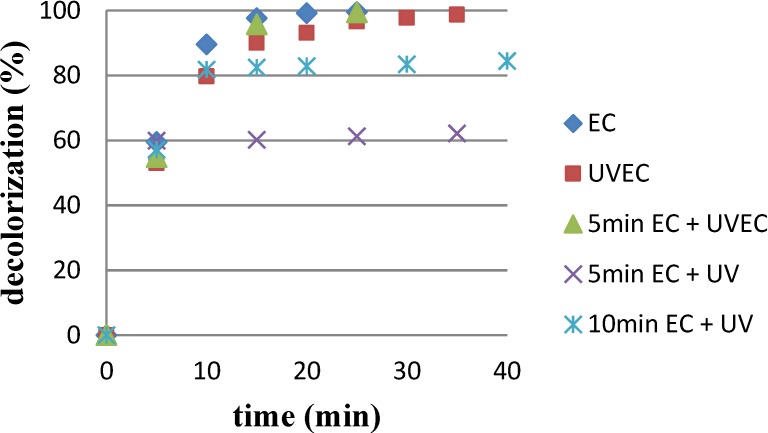
PN effluent decolorization evolution with different combination of electrochemical (10 A) and photo-electrochemical treatments.

As can be seen in Figure 2, the decolorization value was stabilized after a certain treatment time. From that moment, the decolorization value is not enhanced when the treatment time is increased.

**Figure 3:**
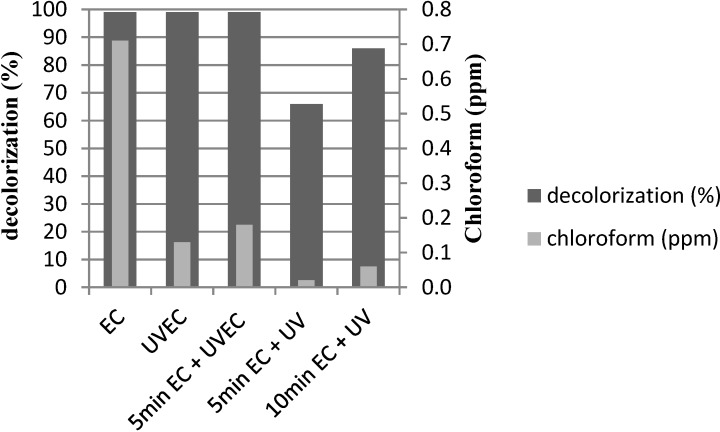
Final decolorization of PN effluent and chloroform generated *versus* the different electrochemical (10 A) and photo-electrochemical treatments.

The samples for haloforms detection were collected when the higher decolorization value was achieved. Chloroform was the unique halogenated compound detected by the GCMS analysis. The concentration of this compound generated in the different treatments is plotted in Figure 3.

According to Figure 3, it can be seen that the electrochemical treatment alone is able to achieve 99% decolorization, but at the same time, 0.7 ppm of chloroform are generated. A clear reduction of this concentration is achieved with the UV radiation treatments, both applied simultaneously during the electrochemical treatment or after it, as a final treatment. UV treatment is able to reduce chloroform concentration below 0.2 ppm.

Taking into account all the results obtained, including decolorization and chloroform removal, we consider that the best method is the trial E, where the electrochemical treatment was applied until 80% of decolorization, with posterior UV radiation (10 min EC + UV). This procedure achieved 86% effluent decolorization (which is already an acceptable value for the subsequent discharge). Under these conditions, the final chloroform concentration was lower than 0.1 ppm and the final dye concentration was lower than 0.5 ppm, which is acceptable for the reuse step. In addition, in the case of effluent discharge, this concentration is not appreciated by the human eye after a 1/10 dilution which corresponds to the mixture of the dyeing bath with the rest of effluents (rinsing and washing baths).

### 2.3. Application of the Optimized Photo-Electrochemical Treatment to Nine Reactive Dyes

The photo-electrochemical treatment optimized in the previous steps was applied to nine reactive dyes (described in Section 3.1). Solutions were prepared as indicated in Section 3.2 to simulate a dyeing effluent. In Table 2 are summarized the kinetic rates, total organic carbon (TOC) removal and the chloroform concentration for each dyeing effluent.

**Table 2:** Optimized electrochemical and photo-electrochemical treatments applied to 9 reactive dyes: kinetic rate, TOC removal and chloroform generation.

Dye	Kinetic rate (min^−1^)	*R*^2^	TOC removal (%)	Chloroform (ppm) [EC]	Chloroform (ppm) [EC + UV]
PMX2R	0.1795	0.9919	12	0.57	0.05
PC	0.1162	0.9998	6.81	1.66	0.14
PY	0.0833	0.9963	11.86	0.65	0.05
PB	0.2622	0.9907	8.33	0.09	0.01
PN	0.2686	0.9954	9.78	0.70	0.06
RB5	0.2471	0.9927	4.31	0.78	0.07
CD	0.1301	0.9985	7.84	0.45	0.04
CR	0.1593	0.9975	9.38	0.57	0.05
CA	0.1295	0.9973	7.04	0.33	0.03

No tendency is detected with respect to the number of functional groups in the dye molecule. In all cases, dyes degradation follows a first order kinetic rate. In addition, in all the studied cases, 86% decolorization was achieved. The TOC removal values are poor, in a range from 4% to 11% depending on the dye. However, recent studies [8] with more concentrated effluents showed that the TOC removal is dependent on dye concentration. 50%–80% TOC removal can be achieved when the dye concentration is 1 g/L or when the treatment time is increased. The authors consider that the organic matter removal is not the goal of this work and consequently, an extended electrochemical treatment is not necessary. There are more efficient methods for dye mineralization, from the economic point of view, such as biological plants. Thus, the aim of this study is the decolorization of the effluent and its further reuse or discharge to a biological plant (where the total mineralization is carried out). In Section 2.4, the influence of TOC removal in the effluent reuse for a new dyeing process is studied. In addition, the chloroform concentration was analyzed after the EC and the EC + UV treatment. As expected, the combination of both techniques (electrochemistry and UV irradiation) reduces the amount of chloroform. The chloroform concentrations detected after the EC + UV treatment were lower than 0.1 ppm, except for PC, for which the value was 0.14 ppm. On average, 90% of chloroform reduction is obtained in the EC + UV treatment with respect to EC treatment.

### 2.4. Reuse of the Decolorized Effluents

The decolorized effluents were reused in a new dyeing process. The color differences (calculated as detailed in Section 3.6) were evaluated with the parameter DE_CMC (2:1)_ with respect to a reference dyeing. In all the studied cases, these values were lower than 1, which is the acceptance limit for the textile industry (Table 3). This implies that all the new dyeings are within the acceptance range. Consequently, the dyes mineralization is not necessary for the effluent reuse because a shorter decolorization treatment provide satisfactory results.

The reuse procedure enables saving 70% of the dyeing water and leads a considerable reduction of effluents salinity. Hence, the photo-electrochemical technique described can be considered an ecofriendly method for the treatment and reuse of dyeing effluents.

**Table 3:** Color differences: fabrics dyed with the treated and reused effluent *vs.* a reference dyeing.

Dye	DL_CMC_	DC_CMC_	DH_CMC_	DE_CMC (2:1)_
**PMX2R**	0.36	−0.26	0.10	0.46
**PC**	0.06	−0.14	−0.07	0.16
**PN**	−0.43	0.13	0.22	0.50
**PY**	−0.42	−0.21	−0.33	0.57
**PB**	0.04	−0.23	0.04	0.24
**RB5**	−0.05	0.36	0.03	0.36
**CD**	−0.51	−0.20	0.11	0.56
**CR**	0.04	0.05	0.09	0.11
**CA**	0.01	0.04	−0.01	0.05

In order to evaluate the influence of the residual organic matter in the dyeing color differences, longer electrochemical treatments were applied to the PN effluent. Higher TOC degradations were obtained, and the decolorized effluents were reused in new dyeing processes. The dyeings performed with these reused baths showed the color differences (*versus* a reference) listed in Table 4.

**Table 4:** Influence of the TOC removal in the PN dyeing result. Color differences of fabrics dyed with the treated and reused effluent *vs.* the reference.

TOC removal (%)	DE_CMC (2:1)_	DL_CMC_	DC_CMC_	DH_CMC_
4.74	0.6	−0.42	0.09	0.42
5.97	0.59	0.38	0.40	0.22
7.21	0.48	0.02	0.34	0.34
11.91	0.37	0.14	0.23	0.24

**Figure 4:**
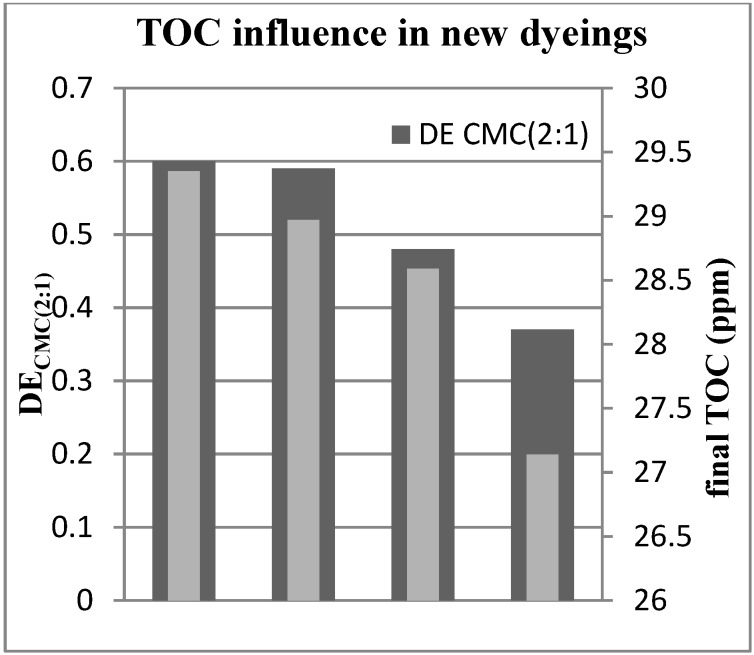
Influence of TOC removal in the reuse of treated effluent for PN: color differences (DE_CMC (2:1)_) of dyeings performed with different TOC content effluents.

This table and Figure 4 showed a clear relationship between color differences and the organic matter removal. The higher the TOC removal, the lower the color differences. However, it must be underlined that in all the studied cases, the color differences are into the limits of acceptance for the industry. For this reason, it is not necessary to increase the TOC removal rate as an extended electrochemical treatment would imply higher power consumption.

In addition, it can be stated that all dyes behave in a similar way. Then, the number of functional groups is not an influent parameter in the reuse process.

## 3. Experimental Procedures

### 3.1. Dyes and Reagents

Nine reactive dyes, including monofunctional, bifunctional and trifunctional structures, were kindly provided by DyStar. Their name and functional groups are listed in Table 5.

**Table 5:** Description of selected Reactive Dyes.

Abbr.	Commercial name	C.I. name	Chromophore	Reactive groups	Type of Reactive group	λ_max_ (nm)
**PN**	Procion Navy H-EXL	Not registered	Disazo	2	monochlorotriazine	606
**RB5**	Remazol Black 133B	Reactive Black 5	Disazo	2	sulphatoethylsulfone	598
**PMX2R**	Procion Orange MX-2R	Reactive Orange 4	Monoazo	1	dichlorotriazine	489
**PY**	Procion Yellow H-EXL	Reactive Yellow 138:1	Disazo	2	monochlorotriazine	416
**PC**	Procion Crimson H-EXL	Reactive Red 231	Disazo	2	monochlorotriazine	545
**PB**	Procion Blue H-EXL	Not registered	Not known	2	Not known	624
**CD**	Cibacron Deepnight S-R	Not registered	Not known	3	Not known	583
**CR**	Cibacron Ruby S-3B	Not registered	Not known	3	Not known	543
**CA**	Cibacron Yellow S-3R	Not registered	Not known	3	Not known	417

**Figure 5:**
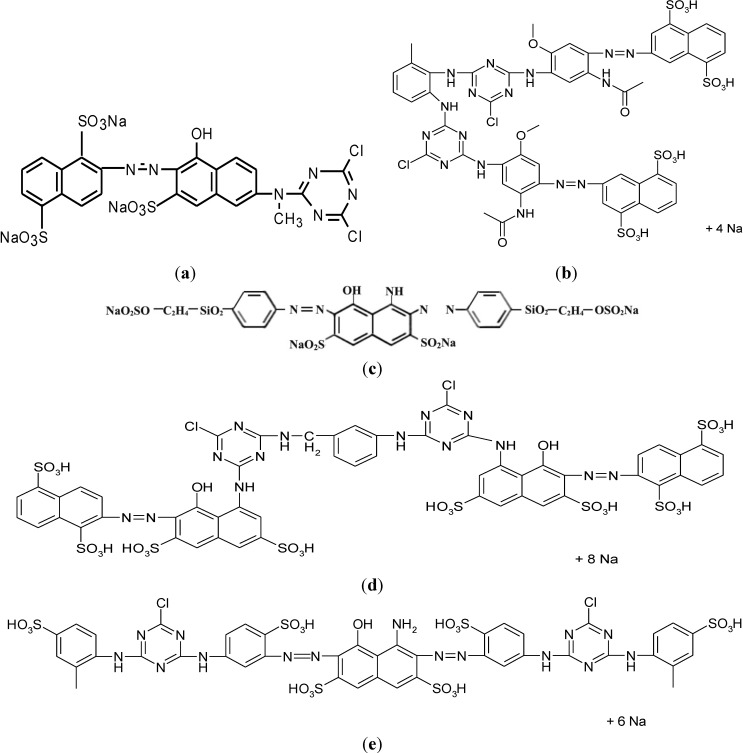
Chemical structure of (**a**) PMX2R; (**b**) PY; (**c**) RB5; (**d**) PC; (**e**) PN.

Figure 5 shows their chemical structure. The formula corresponding to PB, CD, CR, CA have not already been published.

Dyeing was performed on 100% cotton fabrics, kindly provided by TIPSA.

The chemical products used in the effluents and the dyebath preparation (NaOH, Na_2_SO_4_, and NaCl) were of analysis quality supplied by Merck and Fluka (Madrid, Spain). The Internal Standard for the gas chromatography was p-Bromofluorobenzene chromatography grade provided by AccuStandard (Barcelona, Spain).

### 3.2. Effluent Preparation

Dyebath effluents were prepared in the laboratory to simulate an industrial effluent after the dyeing process with reactive dyes. During the dyeing process, the dye unfixed to the fiber remains hydrolyzed in the bath solution as the reactive groups of the dye molecule were replaced by OH groups. The effluents containing 0.1 g/L of hydrolyzed reactive dyes were prepared previously to the electrochemical treatment. The hydrolysis was carried out by heating at 80 °C for 2 h at pH 12 with 0.01 M·NaOH solution (about 10% of the total dyebath volume).

As indicated, chloride plays an important role in dyes indirect oxidation. For this reason, sodium chloride (0.3 g/L) was added to simulate the concentration of tap water (industrial dyeings are performed with decalcified water). The final conductivity of the simulated effluent was adjusted to 35 mS/cm with sodium sulfate in order to reach the industrial effluents conductivity.

### 3.3. Electrochemical Treatment

**Figure 6:**
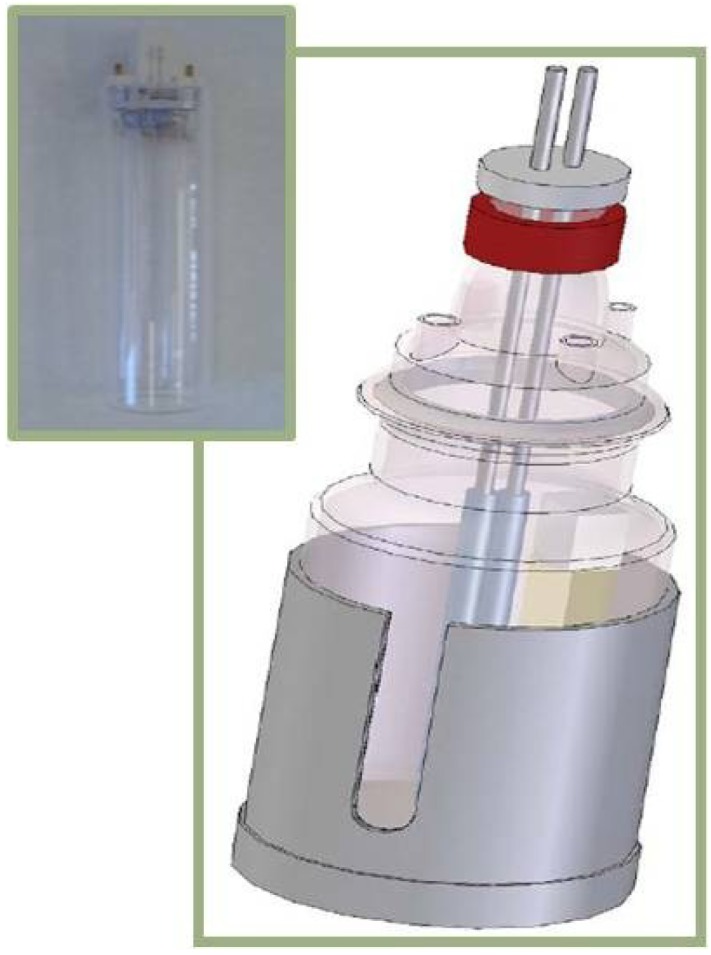
Electrolytic cell with the UV source.

The synthetic effluents were treated in an undividable electrolytic cell, showed in Figure 6.

In all cases, the treated volume was 2 L and studies were carried out under galvanostatic conditions, with a power supply Grelco GVD310 0–30 Vcc/0–10 A (Cornellà de Llobregat, Barcelona, Spain) at intensities 2 A, 5 A and 10 A. The cathode was made of stainless steel and the anode was a DSA (Dimensionally Stable Anode) made of Ti/Pt, with 59.32 cm^2^ of active surface.

The UV radiation was performed with a Philips TUV lamp PL-S UV-C (Sanchinarro, Madrid, Spain) at 254 nm and 9 watts.

### 3.4. Effluent Reuse

Dyeing tests were performed with a TICOLOR instrument under the following conditions: 10 g of cotton fabric, dyestuff concentration 3% o.w.f (on the weight of fiber), liquor ratio 1/10 and 35 g/L of Na_2_SO_4_. NaOH was used as dyeing alkali (until pH 12).

According to the information supplied by DyStar [28], the dyeing method “all in” was selected. The dye and the entire electrolyte were included in the initial dyeing bath and the alkali was added when the dyeing process was already started. The dyeing started at 50 °C for 15 min, then the temperature was raised to 80 °C at a gradient of 1.4 °C/min. The alkali was added after 30 min at 80 °C and the dyeing lasted for 60 min more.

All the experiments were run in triplicate.

After the dyeing process, a washing process takes place where the cotton dyed fabrics were washed to eliminate the dye not fixed to the fabric. This process consists on nine successive washes at different temperatures. The first ones (1st–3rd washes) were at 50 °C with tap water during 10 min, then a soaping step (4th wash) took place with 2 g/L of COTEMOLL TLTR at 95 °C during 15 min. Then, a tap water step (5th wash) and another soap step (6th wash) were carried out with the same conditions described above. Finally, another three water washes at 50 °C were carried out during 10 min (7th–9th washes). All steps were performed at liquor ratio 1:10.

### 3.5. Analyses and Instruments

#### 3.5.1. Spectroscopic Analysis

The decolorization process was studied by spectroscopy analysis, where the initial dyes absorbance (Abs_0_) was compared with the absorbance of the samples collected during the treatment (Abs_t_). The absorbance was measured at the visible maximum dye absorption wavelength (606 nm for PN, 598 nm for RB5, 489 nm PMX2R, 416 nm for PY, 545 nm for PC, 624 nm for PB, 583 nm for CD, 543 nm for CR and 417 nm for CA). Samples were collected each 5min during the electrochemical treatment, and the decolorization was reported in % (Equation (6)).

(6) $$\text{D}\left(\text{\%}\right)=\frac{({\text{Abs}}_{0}-{\text{Abs}}_{\text{t}})\times 100}{{\text{Abs}}_{0}}$$

Absorbance measurements were carried out with a UV-Vis spectrophotometer (Shimadzu UV-2401 PC, Kyoto, Japan).

#### 3.5.2. Kinetic Evolution

In accordance with previous studies [13], the dyes degradation with the electrochemical treatment follows a first-order reaction. The decolorization rate constants (K_d_) were calculated from the slope of semilogarithmic absorbance values rate *versus* exposition time (t) or charge (Q), in accordance with the kinetic Equations (7)–(9):

(7) $$-\text{ln}\frac{{\text{Abs}}_{\text{t}}}{{\text{Abs}}_{0}}={\text{K}}_{\text{d}}\times \text{t}$$

(8) $$-\text{ln}\frac{{\text{Abs}}_{\text{t}}}{{\text{Abs}}_{0}}={\text{K}}_{\text{d}\prime }\times \text{Q}$$

where:

(9) $$\text{Q}\left(\frac{\text{A}\cdot \text{h}}{\text{L}}\right)=\left(\frac{\text{I}\times ({\text{t}}_{\text{t}}-{\text{t}}_{\text{t}-1})}{\text{vol}}\right)$$

#### 3.5.3. TOC Removal

The Total Organic Carbon was determined with a Shimadzu TOC5050A instrument. The TOC degradation was reported in % (see Equation (10)).

(10) $$\text{TOC removal}\left(\text{\%}\right)=\frac{({\text{TOC}}_{0}-{\text{TOC}}_{\text{t}})\times 100}{{\text{TOC}}_{0}}$$

#### 3.5.4. GCMS Analysis

In order to evaluate the possible generation of volatile halogenated compounds, a GCMS method was used for the identification of 23 halogenated compounds listed by the Environmental Protection Agency (EPA-624 method) [29]. An Internal Standard (IS), p-bromofluorobenzene, was used for the quantification of all these compounds.

The gas chromatography analyses were carried out in a Shimadzu QP 2010 (GCMS) system with a Mass Spectrum detector. The sample injection was carried out by a headspace technique, where 15 mL of the sample was heated to 80 °C for 45 min in a 20 mL vial. The vial was sealed with silicone/PFTE septum and 1 mL of gas was injected to the GCMS. The injection temperature was 200 °C. The chromatography program started at 35 °C during 10 min, the gradient rate was increased at a rate of 4 °C/min until it reached 150 °C, which is the final temperature (10 min). The column selected was TRB-624 (length 30 m, internal diameter 0.25 mm and 1.4 µm packing). The carrier gas was Helium at a 0.95 mL/min column flow rate.

The identification of halogenated compounds was performed using Nist 147, Nist 27 and Wiley 229 as reference libraries. Compounds, detection limits and the retention times are listed in Table S1 of Supporting information.

The quantitative analysis is carried out by using the Equation (11):

(11) $${\text{Concentration}}_{\text{c}}=\frac{{\text{Area}}_{\text{c}}\times {\text{Concentration}}_{\text{IS}}}{{\text{Area}}_{\text{IS}}\times {\text{R}}_{\text{f}}}$$

where R_f_ is the response factor, which is specific for each compound. It corresponds to the rate between the chromatographic response of the halogenated compound detected and the Internal Standard (1-bromo, 3-fluorobenzene).

### 3.6. Color Evaluation

The final dyed fabric color was measured by a spectrophotometer Macbeth Color Eye 7000, with illuminate D65 and 10° of standard observer. The instrument evaluates the chromatic coordinates of each dyed fabric. These coordinates are defined by three parameters (Lightness DL_cmc_; Chrome DC_cmc_, and Hue DH_cmc_) which compromise the color difference between the standard and the reused effluent dyeing. The color difference with the formula DE_CMC (2:1)_ is represented in the Equation (12) according to UNE-EN ISO 105-J03: 1997 [30].

DE_CMC (2:1)_ = [(DL^*^/2S_L_)^2^ + (DC^*^_ab_/S_c_)^2^ + (DH^*^_ab_/S_H_)^2^]^1/2^ (12)

where:

S_L_ = 0.040975L^*^_R_/(1 + 0.01765L^*^_R_) if L^*^_R_ ≥ 16;
Or S_L_ = 0.511 if L^*^_R_ < 16;
S_c_ = [0.0638 C^*^_ab,R_/(1 + 0.0131C^*^_ab,R_)] + 0.638;
S_H_ = (FT + 1 − F) S_c_;

and:

F = {(C^*^_ab,R_)^4^/[(C^*^_ab,R_)^4^ + 1900]}^1/2^;
T = 0.36 + |0.4cos(35 + h_ab,R_)| if h_ab,R_ ≥ 345° or h_ab,R_ ≤ 164°;
Or T = 0.56 + |0.2 cos(168 + h_ab,R_)| if 164° < h_ab,R_ < 345°.

In general, the acceptance limit for color differences in the textile industry is one unit (DE_CMC (2:1)_ ≤ 1). This criterion is widely used in dyeing quality control to compare the color differences between two fabric samples.

## 4. Conclusions

The electrochemical treatment achieved 99% of dye decolorization at all the studied intensities, although it was more efficient at the higher current intensity, 10 A. When this technique was applied with simultaneous UV irradiation, the decolorization kinetic rates decreased, whereas dye decolorization was enhanced by the application of UV after the electrochemical treatment. In any case, the UV irradiation reduced the chloroform content.

The most efficient combination to treat reactive dye effluents is the electrochemical treatment up to 80% decolorization followed by UV irradiation. In this way, the chloroform concentration was lower than 0.1 ppm except for PMX-2R. No influence of the number of functional groups in the dye molecule was evidenced.

It was also demonstrated that the complete dye mineralization was not necessary to reuse the treated effluent in new dyeing processes. Decolorized effluents with only 4% TOC removal provided color differences into the acceptable range (DE_CMC (2:1)_ < 1), which resulted in reducing significantly effluent salinity and saving 70% water.

## Supplementary Materials

Supplementary materials can be accessed at: http://www.mdpi.com/1996-1944/7/11/7349/s1.
