# Supplementary material for: Photo-Electrochemical Treatment of Reactive Dyes in Wastewater and Reuse of the Effluent: Method Optimization
Source: Materials (Basel). 2014 Nov 14;7(11):7349–65. doi: 10.3390/ma7117349 (PMC5512639; doi:10.3390/ma7117349)
Supplement: Supplementary file 1 [file materials-07-07349-s001.pdf]

Supporting Information

Table S1. Volatile halogenated compounds selected for the identification and quantification.

Compound	CAS number	D.L. (μg/L)	Retention time (min)
Bromomethane	74-83-9	10	2.50
Chloroethane	75-00-3	1.0	2.65
Trichlorofluoromethane	75-69-4	2.5	3.00
1,1- Dichloroethene	75-35-4	2.5	3.86
Diclhoromethane	75-09-2	1.0	4.90
1,1-Dichloroethane	75-34-3	2.5	6.55
Trichloromethane	67-66-3	1.0	9.69
1,1,1-Trichloroethane	71-55-6	2.5	10.07
Tetrachloromethane	56-23-5	2.5	10.63
Benzene	71-43-2	2.5	11.67
1,2-Dichloroethane	107-06-2	10	12.02
Trichloroethene	79-01-6	1.0	14.29
Bromodichloromethane	75-27-4	5	16.52
c-1,3-Dichloropropene	10061-01-5	10	18.22
Tetrachloroethene	127-18-4	1.0	21.03
Dibromoclhoromethane	124-48-1	10	22.32
Chlorobenzene	108-90-7	1.0	24.32
Ethyl-benzene	100-41-4	1.0	24.82
Tribromomethane	75-25-2	10	27.45
1-bromo,3-fluorobenzene	1073-06-9	2.5	28.61
1,3-Dichlorobenzene	541-73-1	1.0	32.48
1,4-Dichlorobenzene	106-46-7	0.5	32.85
1,2-Dichlorobenzene	95-50-1	0.5	34.17
